# Protective Effects of PEP-1-GSTA2 Protein in Hippocampal Neuronal Cell Damage Induced by Oxidative Stress

**DOI:** 10.3390/ijms24032767

**Published:** 2023-02-01

**Authors:** Yeon Joo Choi, Min Jea Shin, Gi Soo Youn, Jung Hwan Park, Hyeon Ji Yeo, Eun Ji Yeo, Hyun Jung Kwon, Lee Re Lee, Na Yeon Kim, Su Yeon Kwon, Hyo Young Jung, Yong-Jun Cho, Dae Won Kim, Jinseu Park, Kyu Hyung Han, Keun Wook Lee, Jong Kook Park, Chan Hee Lee, Won Sik Eum, Soo Young Choi

**Affiliations:** 1 Department of Biomedical Science, Research Institute of Bioscience & Biotechnology, Hallym University, Chuncheon 24252, Republic of Korea; 2 Department of Veterinary Medicine, Institute of Veterinary Science, Chungnam National University, Daejeon 34134, Republic of Korea; 3 Department of Neurosurgery, Hallym University Medical Center, Chuncheon 24253, Republic of Korea; 4 Department of Biochemistry and Molecular Biology, Research Institute of Oral Sciences, College of Dentistry, Gangneung-Wonju National University, Gangneung 25457, Republic of Korea

**Keywords:** ischemia, PEP-1-GSTA2, oxidative stress, neuroprotection, protein therapy

## Abstract

Glutathione S-transferase alpha 2 (GSTA2), a member of the glutathione S-transferase family, plays the role of cellular detoxification against oxidative stress. Although oxidative stress is related to ischemic injury, the role of GSTA2 against ischemia has not been elucidated. Thus, we studied whether GSTA2 prevents ischemic injury by using the PEP-1-GSTA2 protein which has a cell-permeable protein transduction domain. We revealed that cell-permeable PEP-1-GSTA2 transduced into HT-22 cells and markedly protected cell death via the inhibition of reactive oxygen species (ROS) production and DNA damage induced by oxidative stress. Additionally, transduced PEP-1-GSTA2 promoted mitogen-activated protein kinase (MAPK), and nuclear factor-kappaB (NF-κB) activation. Furthermore, PEP-1-GSTA2 regulated Bcl-2, Bax, cleaved Caspase-3 and -9 expression protein levels. An in vivo ischemic animal model, PEP-1-GSTA2, markedly prevented the loss of hippocampal neurons and reduced the activation of microglia and astrocytes. These findings indicate that PEP-1-GSTA2 suppresses hippocampal cell death by regulating the MAPK and apoptotic signaling pathways. Therefore, we suggest that PEP-1-GSTA2 will help to develop the therapies for oxidative-stress-induced ischemic injury.

## 1. Introduction

The multifunctional protein Glutathione S-transferase (GST) plays a key role in cellular detoxification and signal transduction [1,2]. Mammalian cytosolic GSTs have been identified such as alpha, theta, zeta, omega, sigma, pi and mu, and alpha class GSTs are the most abundant in mammalians [3,4]. Among the alpha classes, GST alpha 1 (GSTA1) and GST alpha 2 (GSTA2) are highly expressed in the liver and breast [5,6]. 

GSTA2 has a protective function against oxidative stress as one of the functional antioxidant response elements and Bousova et al. reported that the overexpression of GSTA2 showed a compensatory function against depending on the elevation of oxidative stress, whereas a reduction in GSTA2 impaired the defense system against oxidative stress [7,8]. Additionally, several studies have shown that GSTA2 inhibited the cell death of human erythroleukemia and lens epithelial cells, and these reports indicate that GSTA2 plays a key role in cellular detoxification under oxidative stress [9,10,11,12,13]. 

ROS are overexpressed under oxidative stress which leads to cell death due to lipid peroxidation, DNA, carbohydrate and protein damages [14,15,16], and the overproduction of ROS is related to neuronal diseases via the reaction with the apoptotic cell signaling pathway [17,18,19,20]. MAPK signaling is activated by ROS and involved in cell survival and apoptosis [21,22,23,24]. Therefore, it is important to study the regulation of oxidative stress and the MAPK signaling pathways for protecting neuronal cell damage. 

The protein transduction domain’s (PTD) ability to deliver proteins into cells across the cell membrane and blood–brain barrier (BBB) without toxicity [25,26] was explored, and various PTD-attached proteins were used to investigate the functional roles [25,26,27,28,29,30,31,32,33,34,35]. We showed that the cell-permeable PEP-1-GSTA2 fusion protein protected against hippocampal neuronal cell death in HT-22 cells and an ischemic animal model. 

## 2. Results

### 2.1. Preparation and Cell Permeation of Fusion Protein

**Figure 1:**
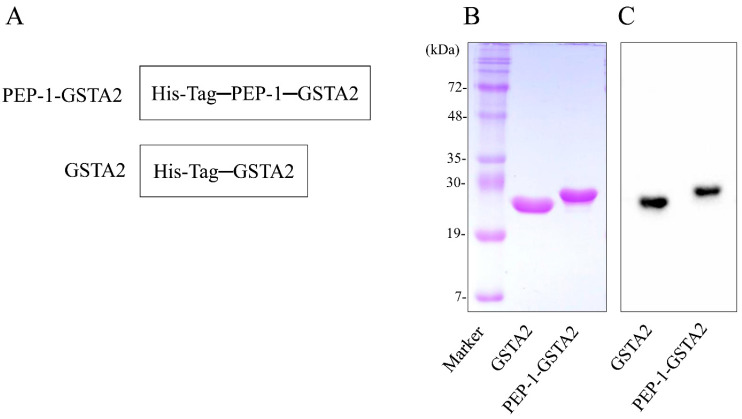
Purification of PEP-1-GSTA2 protein. Diagrams of PEP-1-GSTA2 and control GSTA2 proteins (**A**). Purified PEP-1-GSTA2 and control GSTA2 proteins were identified by 15% SDS-PAGE (**B**) and were detected via Western blotting using an anti-histidine antibody (**C**).

To construct PEP-1-GSTA2 plasmid, the human GSTA2 gene was cloned in the PEP-1 expression vector. Figure 1A shows that the PEP-1-GSTA2 expression vector consists of six histidine, PEP-1-peptide and cDNA of human GSTA2. After transformation of the PEP-1-GSTA2 plasmid, overexpressed protein was purified and confirmed (Figure 1B,C).

**Figure 2:**
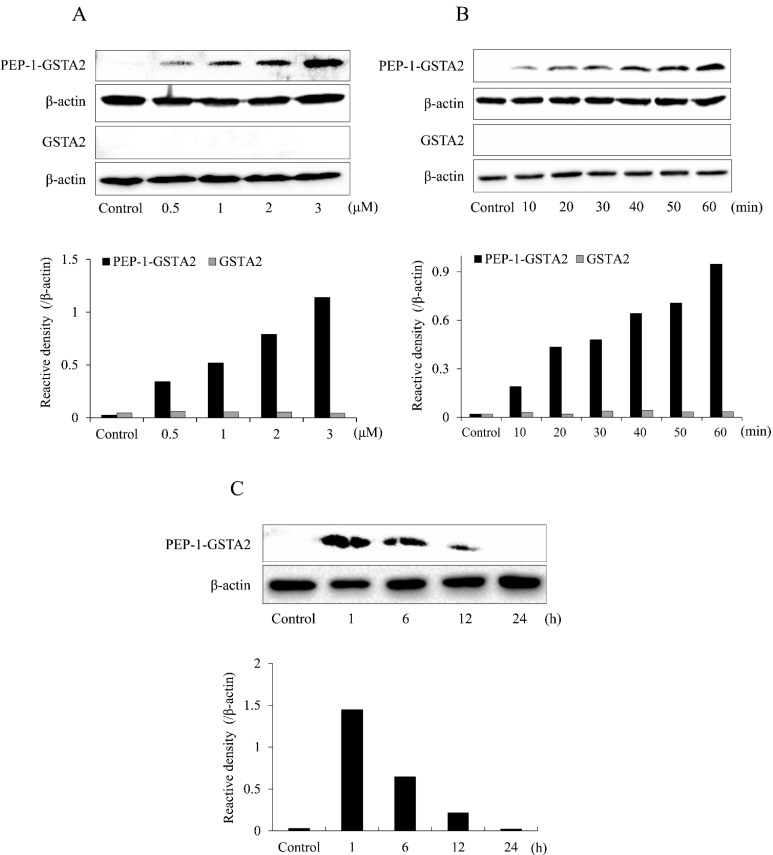
Transduction of PEP-1-GSTA2 proteins into HT-22 cells. The cell culture media were treated with PEP-1-GSTA2 protein at different doses (0.5–3 μM) or control GSTA2 protein for 1 h (**A**). The cell culture media were treated with PEP-1-GSTA2 proteins (3 μM) or control GSTA2 protein for different time periods (10–60 min) (**B**). Intracellular stability of transduced PEP-1-GSTA2 protein. HT-22 cell culture media were incubated for 24 h after transduction of PEP-1-GSTA2 protein for 1 h (**C**). Then, transduction of PEP-1-GSTA2 protein was assessed via Western blotting using an anti-histidine antibody and the intensity of the bands was measured using a densitometer.

**Figure 3:**
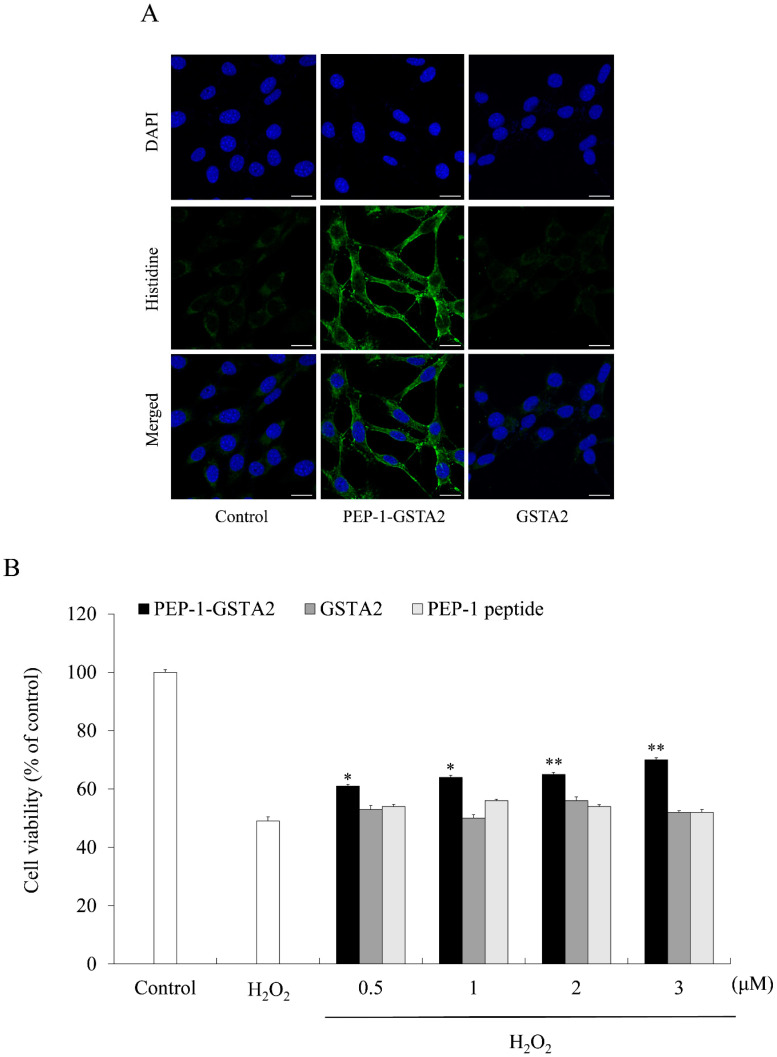
Effects of transduced PEP-1-GSTA2 protein against H_2_O_2_-induced cell viability. HT-22 cell culture media were treated with PEP-1-GSTA2 protein (3 μM) or control GSTA2 protein for 1 h. Cellular localization of transduced PEP-1-GSTA2 proteins was confirmed via fluorescence microscopy (**A**). Scale bar = 20 μm. Effect of transduced PEP-1-GSTA2 protein on cell viability. HT-22 cells were pretreated with PEP-1-GSTA2 protein or control GSTA2 (0.5–3 μM) for 1 h and exposed to H_2_O_2_ (600 μM) for 2 h. Then, cell viability was assessed via MTT assay (**B**). * *p* < 0.05 and ** *p* < 0.01 compared with H_2_O_2_-treated cells.

To assess whether PEP-1-GSTA2 could transduce into cells, HT-22 cells were treated with different concentrations (0.5–3 μM) for 60 min of PEP-1-GSTA2 or for different times (10–60 min) of PEP-1-GSTA2 (3 μM), as shown in Western blot analysis presented in Figure 2. The PEP-1-GSTA2 protein transduces HT-22 cells and existed for up to 12 h, while no band was detected in the cells treated with GSTA2 protein without PEP-1 peptide. Additionally, the distributions of PEP-1-GSTA2 in the cells were determined using immunofluorescence staining (Figure 3A). Extensive green fluorescence was observed in both the cytoplasm and nucleus in the PEP-1-GSTA2-treated cells. 

### 2.2. Effects of PEP-1-GSTA2 on Oxidative Stress

To evaluate the effects of PEP-1-GSTA2 on cell viability, cell viability was determined using an MTT assay. Figure 3B showed that being exposed only to H_2_O_2_ markedly reduced HT-22 cell viability to 49% of control cells. However, treatment with PEP-1-GSTA2 increased cell viability up to 70%, while treatment with GSTA2 had no effect at all. 

**Figure 4:**
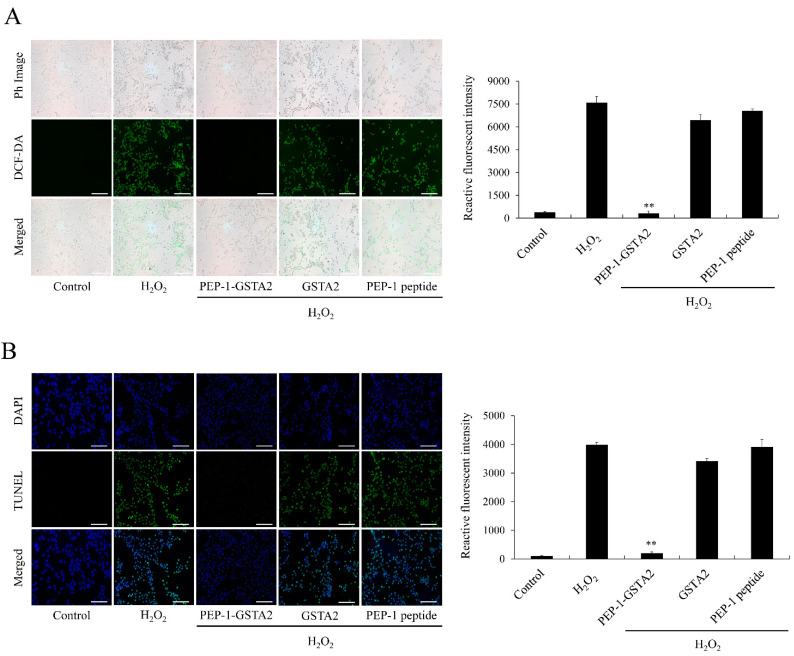
Effects of PEP-1-GSTA2 protein against H_2_O_2_-induced ROS production and DNA fragmentation. HT-22 cells were treated with PEP-1-GSTA2 protein (3 μM) or control GSTA2 protein for 1 h before treatment with 600 μM H_2_O_2_ for 1 h or 4 h. Then, intracellular ROS levels (**A**) and DNA fragmentation (**B**) were determined via DCF-DA and TUNEL staining. Fluorescence intensity was quantified using an ELISA plate reader. Scale bar = 50 μm. ** *p* < 0.01 compared with H_2_O_2_-treated cells.

In order to investigate the effects of PEP-1-GSTA2 on ROS production and DNA damage in cells, DCF-DA and TUNEL staining were performed. The results showed that ROS and DNA damage were reduced after treatment of PEP-1-GSTA2, while treatment with GSTA2 had no effect (Figure 4).

### 2.3. PEP-1-GSTA2-Activated Phosphorylation of Akt, MAPKs and p65

**Figure 5:**
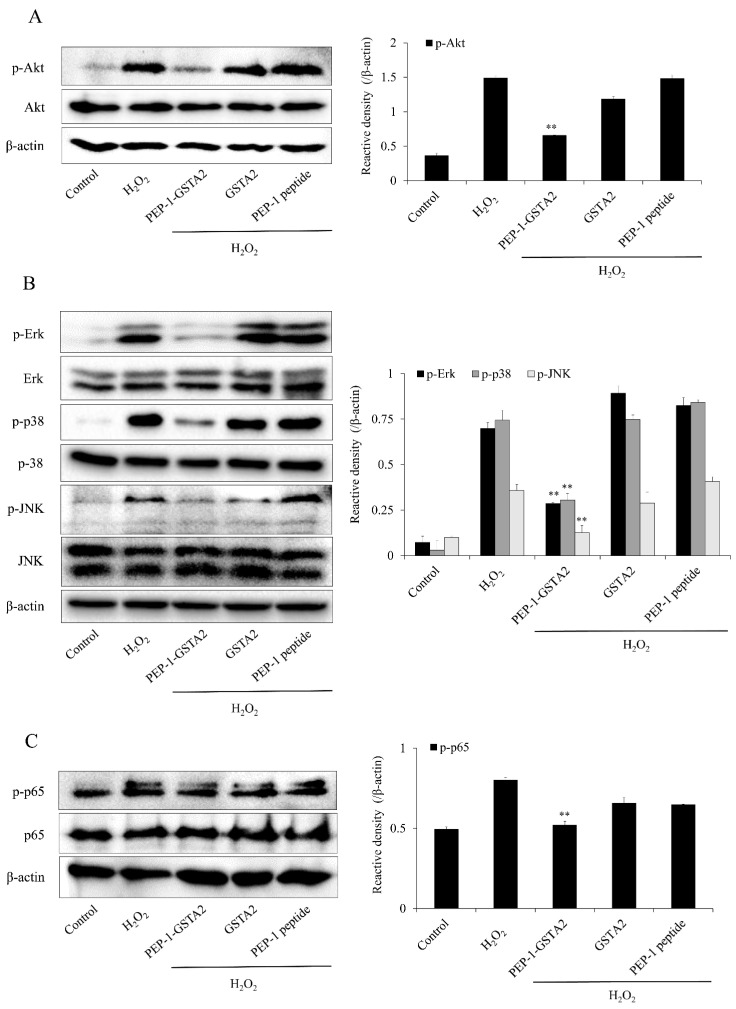
Effects of PEP-1-GSTA2 protein on H_2_O_2_-induced MAPK activation in HT-22 cells. The cells were treated with PEP-1-GSTA2 protein (3 μM) or control GSTA2 protein for 1 h before being exposed to H_2_O_2_ (600 μM). Akt (**A**), MAPK (**B**) and NF-κB (**C**) activation were analyzed via Western blotting. Band intensity was measured using a densitometer. ** *p* < 0.01, compared with H_2_O_2_-treated cells.

MAPKs and NF-κB phosphorylation are highly involved with oxidative-stress-induced ROS and lead to cell death [36,37]. We evaluated the expression of Akt, MAPKs and p65 to determine the signaling mechanism of PEP-1-GSTA2 in HT-22 cells. Akt, MAPKs and p65 phosphorylated levels were increased, whereas PEP-1-GSTA2 significantly reduced those levels in the H_2_O_2_ exposed cells. However, GSTA2 and PEP-1 peptide showed a similar pattern with cells exposed to H_2_O_2_ alone (Figure 5). 

### 2.4. PEP-1-GSTA2 Regulated Bcl-2, Bax, Cleaved Caspase-3 and -9 Expressions

**Figure 6:**
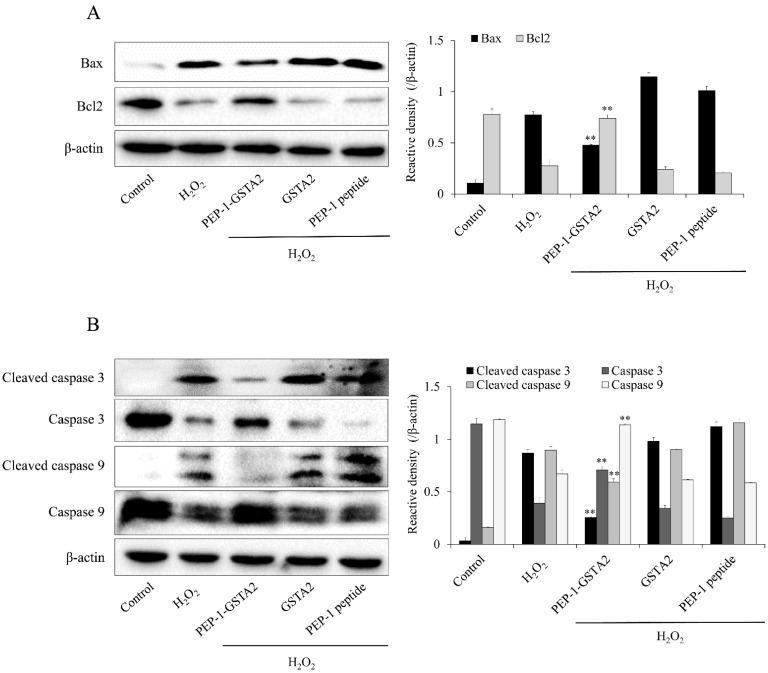
Effects of PEP-1-GSTA2 protein against H_2_O_2_-induced Bcl-2, Bax, Caspase 3 and Caspase 9 expression in HT-22 cells. One hour pretreatment of HT-22 cells with PEP-1-GSTA2 protein (3 μM), control GSTA2 protein (3 μM) or PEP-1 peptide (3 μM) was followed by treatment with H_2_O_2_ (600 μM). The expression levels of Bcl-2 and Bax (**A**), caspase-3 and caspase-9 (**B**) were determined via Western blot analysis and band intensity was measured using a densitometer. ** *p* < 0.01 compared with H_2_O_2_-treated cells.

To further evaluate whether the PEP-1-GSTA2 protein inhibited apoptosis, we assessed the Bax, Bcl-2, cleaved Caspase-3 and -9 expression in cells. As shown in Figure 6, PEP-1-GSTA2 markedly reduced these protein expression levels. In addition, this fusion protein increased the Bcl-2 expression level. However, GSTA2 and PEP-1 peptide had no effect on apoptosis. 

### 2.5. Effects of PEP-1-GSTA2 in an Ischemic Injury Animal Model 

**Figure 7:**
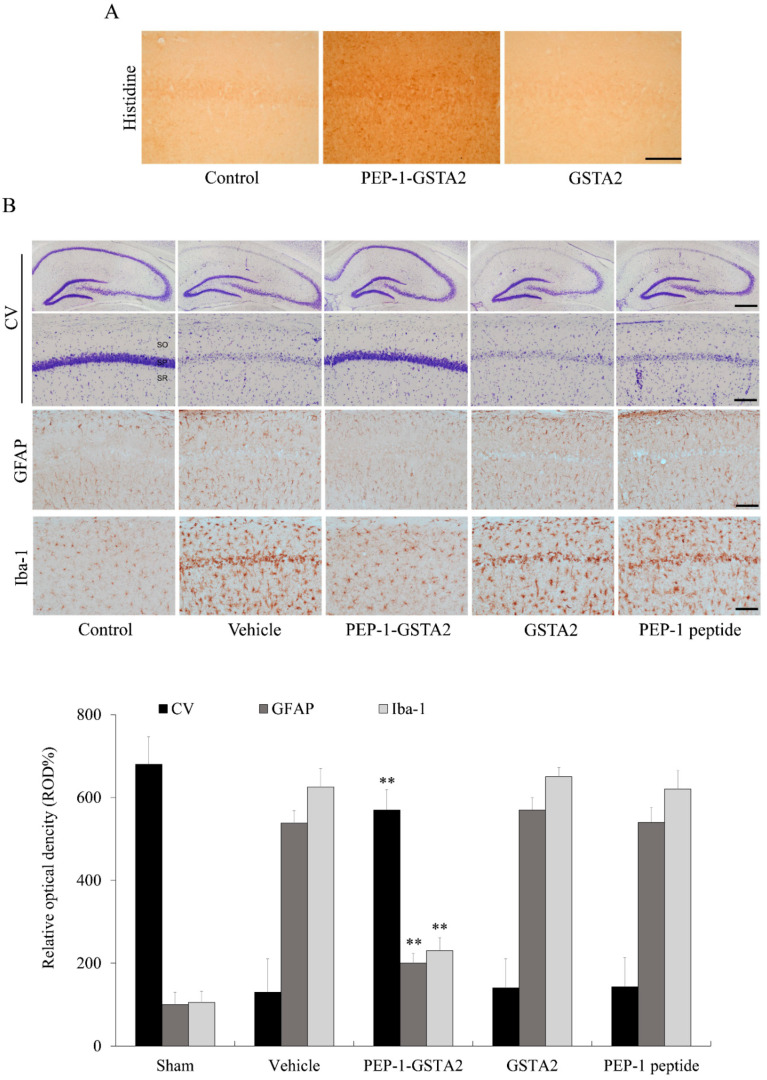
Neuroprotective effects of PEP-1-GSTA2 protein against ischemic damage. Gerbils were treated with single injections of PEP-1-GSTA2 (2 mg/kg) protein and killed after 12 h. Transduction of PEP-1-GSTA2 into the CA1 region of the brain was determined via immunohistochemistry with an anti-histidine antibody (**A**). Scale bar = 100 μm. Gerbils were treated with single injections of PEP-1-GSTA2 (2 mg/kg) protein, control GSTA2 protein or PEP-1 peptide and killed after 7 days. Neuronal cell viability after ischemic insults was determined using CV, GFAP and Iba-1 immunostaining (**B**). Relative numeric analysis of CV, GFAP and Iba-1 positive neurons in the CA1 region. Scale bar = 400 and 50 μm. ** *p* < 0.01, significantly different from the vehicle group.

To investigate whether PEP-1-GSTA2 could protect against an ischemic animal model, an immunostaining experiment was performed. As shown in Figure 7, PEP-1-GSTA2 was transduced into the CA1 region of the brain. However, there was no difference between mice treated with GSTA2 compared to controls. Furthermore, we observed that CV-positive cells were significantly reduced following ischemia–reperfusion, while PEP-1-GSTA2 drastically increased CV-positive cells. 

GFAP and Iba-1 staining showed that neuronal cells were markedly aggregated in the vehicle-treated group, whereas PEP-1-GSTA2 inhibited the aggregation of neuronal cells and similarly displayed the neuronal cells compared with the control group. There were no significant changes in neuronal cells found in GSTA2- and PEP-1-peptide-treated groups compared with the vehicle-treated group.

## 3. Discussion

GSTA2, one of the alpha classes of glutathione S-transferases, is known to be a major defensive protein against oxidative stress and lipid peroxidation [7,8], and this protein is predominantly expressed in the human liver [5,38]. Some reports have demonstrated that an overexpression of GSTA2 protected against cell damage by playing a role in defense under oxidative stress [8,9,10,11,12,13] and that this protein inhibited apoptosis and lipid peroxidation in K562 cells [11]. Recently, Liu et al. reported that capsaicin up-regulated GSTA2 in a Parkinson’s disease animal model and up-regulated GSTA2-inhibited apoptosis and cell death by regulating the autophagy and signaling pathways under oxidative stress. Therefore, the authors suggested that GSTA2 is promising as a therapeutic target for alleviating the progression of PD [39]. Another group also showed that the overexpression of GSTA2 has a protective effect against ROS-induced cell death in hepatocellular carcinoma (HCC) cells [40,41]. Since it is well known that ROS are associated with various diseases, these reports indicate that GSTA2 can be a main therapeutic target because this protein inhibits ROS-induced cell death via glutathione peroxidase activity [21,42,43,44].

The protein transduction domain (PTD) is a powerful tool to deliver the therapeutic protein into cells and tissues without side effects [25,26,45]. PEP-1 is a type of PTD and consists of 21 amino acids. In addition, PEP-1 PTD has some advantages of high stability, efficiency and rapid transduction with low toxicity [46,47]. The efficiency of the transduction of the PTD fusion protein depends on various factors, including the type of PTD, the size of the target protein, and cell types. Additionally, a problem to be solved with non-specific effects in animal experiments is that the intra-tissue delivery of the PTD fusion protein lacks target tissue specificity [26,31]. However, PTD offers an effective tool for transducing therapeutic target proteins into cells or tissues. Therefore, we constructed PEP-1-GSTA2 and showed that this fusion protein protected cell death. In K562 cells, overexpressed GSTA2 protected cells via a reduction in lipid peroxidation and cytotoxic effects in H_2_O_2_-exposed cells [10,11,12], and this protein significantly inhibited cataractogenesis in mice or lens epithelial cells under oxidative stress [48,49]. These reports suggest that GSTA2 has protective roles against the deleterious effects of oxidative stress. 

It is well known that H_2_O_2_ induces neuronal cell death via the activation of Akt and MAPK signaling pathways [50,51]; however, the activation of Akt and MAPK are involved in cell survival in different cell types [52,53,54]. Therefore, we investigated the function of the signaling pathway of PEP-1-GSTA2 in H_2_O_2_-induced HT-22 cells and PEP-1-GSTA2 was shown to suppress Akt and MAPK phosphorylation. Consistent with our results, *Lonicera japonica* THUNB (LJ) suppressed the phosphorylated Akt and MAPKs in SH-SY5Y cells, suggesting that LJ protects against cell death induced by H_2_O_2_ [51]. It has been reported that the phosphorylation of Akt (ser-473) induced by oxidative stress leads to apoptosis and cell death [55]. In addition, the overexpression of GSTA2 in K562 cells caused by transfection significantly reduced JNK activation in H_2_O_2_-exposed cells [11]. 

The apoptotic activator (Bax) and inhibitor (Bcl-2) play important roles in cell death and the activations of Caspase-3 and Caspase-9 are known as makers of apoptotic cell death [56]. It has been reported that GSTs protect Caco-2 cells and hepatocytes cells via the inhibition of oxidative stress and apoptosis [10,57,58]. We also revealed that PEP-1-GSTA2 suppressed cell death via the regulation of apoptotic protein expression levels. 

We further examined the effects of PEP-1-GSTA2 in an ischemic injury animal model. PEP-1-GSTA2 was delivered into the hippocampal CA1 region and protected against cell death in vivo. Other studies have already shown that the cell-permeable PTD fusion protein significantly reduced cell death in an animal model [59,60,61]. It has been reported that the activation of microglial and astrocytes is used as a marker for the detection of ischemic neuronal injury [62,63]. We showed that PEP-1-GSTA2 significantly inhibits ischemic injury by reducing the activation of microglia and astrocytes. The present result demonstrates that PEP-1-GSTA2 plays a protective role to prevent neuronal cell death in ischemia under oxidative stress. 

In conclusion, we have reported here that PEP-1-GSTA2 was transduced into cells in vitro and in vivo and this protein significantly prevented neuronal cell death. Therefore, PEP-1-GSTA2 will help to develop the therapies for neuronal diseases including ischemic injury.

## 4. Materials and Methods

### 4.1. Purification of PEP-1-GSTA2 Proteins 

To obtain the recombinant PEP-1-GSTA2 protein, the cDNA for human GSTA2 was cloned into the pET-15b expression vector as described previously [29]. Briefly, PEP-1-GSTA2 was constructed via PCR. The plasmid PEP-1-GSTA2 was transformed into *E. coli* BL21 (DE3) and induced by adding IPTG (Duchefa, Haarlem, The Netherlands). Then, PEP-1-GSTA2 was purified and the protein concentration was measured as described previously [29,64].

### 4.2. Cell Culture and Transduction of PEP-1-GSTA2 

Cell culture, transduction of fusion protein and Western blot were performed via methods as described in a previous report [28,33,65]. 

### 4.3. Assessment of Cell Viability Using MTT Assay

A protective effect of PEP-1-GSTA2 against H_2_O_2_-induced cell death was determined by measuring the viability of cultured cells using an MTT assay (Abcam, Cambridge, MA, USA) [28,29,66]. The cells were divided as follows: (1) normal control cells; (2) only H_2_O_2_-treated cells; (3) H_2_O_2_ + proteins/peptide (0.5 μΜ)-treated cells; (4) H_2_O_2_ + proteins/peptide (1 μΜ)-treated cells; (5) H_2_O_2_ + proteins/peptide (2 μΜ)-treated cells; and (6) H_2_O_2_ + proteins/peptide (3 μΜ)-treated cells.

HT-22 cells were seeded at a density of 1 × 10^4^ cells per well on a 96-well plate and incubated at 37 °C with 5% CO_2_ for 12 h. PEP-1-GSTA2 (0.5–3 μM) protein was treated for 1 h and washed three times with PBS and trypsin-EDTA. Then, H_2_O_2_ (600 μM) was treated for 6 h and the absorbance was measured at 570 nm using an ELISA microplate reader (Labsystems Multiskan MCC/340, Helsinki, Finland). Cell viability was expressed as a percentage of the normal control cells.

### 4.4. DCF-DA and TUNEL Staining

The levels of ROS and DNA damage were confirmed using DCF-DA (Sigma-Aldrich, St. Louis, MO, USA) and TUNEL (Roche Applied Science, Basel, Switzerland) staining as described in a previous report [28,29,67]. 

### 4.5. Experimental Animals

Male gerbils (65–75 g; 6 months old) were obtained from the Experimental Animal Center at Hallym University. All animal experiments were performed according to the ARRIVE guideline (https://www.nc3rs.org.uk/arrive-guidelines, accessed on July 2020) and approved by the Institutional Animal Care and Use Committee of Soonchunhyang University [SCH16-0004].

The ischemic injury animal models were prepared as described in a previous report [28,30].

### 4.6. Statistical Analysis

All statistical data used GraphPad Prism software (version 5.01; GraphPad Software Inc., San Diego, CA, USA). Values are shown as mean ± standard error of the mean from three experiments. Statistical comparisons between each group were performed using one-way analysis of variance with Bonferroni’s post hoc test. Difference at *p* < 0.05 was considered statistically significant.

## Data Availability

The data presented in this study are available on request from the corresponding author.
